# Evolution and significance of amino acid scores for protein quality

**DOI:** 10.3389/fnut.2024.1437853

**Published:** 2024-09-03

**Authors:** Claire Gaudichon

**Affiliations:** Université Paris-Saclay, AgroParisTech, INRAE, UMR PNCA, Palaiseau, France

**Keywords:** chemical score, amino acids, FAO, PD-CAAS, DIAAS

## Abstract

Amino acid scores have become very popular protein quality scores since their definition and recommendation by FAO expert groups. The chemical score is the central pillar of this method, and has been refined with digestibility correction factors, such as protein digestibility for the PD-CAAS and amino acid digestibility for the DIAAS. Several elements need to be taken into account to properly determine these scores, not only from a methodological point of view but also in order to reconcile regulation, pragmatism, accuracy and also biological significance. This review offers a reminder of the main points raised in the FAO reports on protein and AA requirements in 1995 and 2007, and on protein quality in 1991 and 2013. It also highlights the factors that most impact score metrics, and in particular the choice of reference pattern and protein determination in the food. Lastly, the scores are compared, and versus another quality score based on the physiological response, the protein efficiency ratio.

## Introduction

Amino acid scores have been designed to reflect the ability of dietary protein to satisfy amino acid requirements. They are primarily based on the indispensable amino acid (IAA) content of dietary protein related to human amino acid requirements. They can secondarily include correction factors to account for the digestibility of protein (Protein Digestibility Amino Acid Score, PD-CAAS) or individual amino acids (Digestible Indispensable Amino Acids, DIAAS). A single composite figure resulting from these scores then summarizes this capacity.

## Reference patterns

Amino acid requirements have evolved since the FAO reports in 1985 (1) and 2007 (2) after methods based on the oxidation of ^13^C amino acids were recognized as being more accurate than the N balance method, leading to values up to three times higher for some AAs such as lysine. Briefly, the N balance method consists in determining digestive, urinary and miscellaneous N losses in response to various intake of the amino acid which requirement is to be determined (3). The AA requirement is assumed to correspond to the intake for which N intake is equal to N losses (null balance). In the nineties, two tracer methods, namely Direct AA Oxidation (DAAO) (4) and Indirect AA Oxidation (IAAO) (5) emerged. They were based on the intravenous infusion of a ^13^C labeled AA, which oxidation was measured in expired air in response to various intakes of the AA of interest. When the AA intake is adequate, ^13^C oxidation reaches a minimum through a breakpoint that is considered to correspond to the AA requirement. In children, the factorial method is used to determine the maintenance and the growth components of the requirement. These methods have been described in detail in the FAO report in 2007 in which AA requirements for adults were reevaluated on the basis of ^13^C oxidation methods. AA requirement (expressed per body weight unit) decreases rapidly from the age of 0–6 months to 3 years of age after which AA requirements are very similar to those of adults. To generate a so-called reference pattern, AA requirement values are divided by the protein requirement, which in adults has been established as 0.66 g/kg/d, based on N balance studies (2). The resulting reference pattern is then used to calculate the chemical score. Because AA requirement values differed markedly between the 1985 and 2007 FAO expert reports, the reference patterns published in the reports regarding protein quality evaluation in 1991 (6) and 2013 (7) also differed, as shown in Table 1. In 1991, it was recommended that the reference pattern for infants or preschool children aged 2–5 years should be used. In 2013, three reference patterns were proposed, for infants 0–6 months, children 0.5–3 y and individuals older than 3 y, because of the small difference between AA requirements at 3 y and 18 y. When comparing the FAO 1991 pattern for preschool children and that for individuals >3 y from FAO 2013, both being used for adults, the pattern from 2013 was more favorable, particularly for lysine and aromatic AA (Table 1).

**Table 1:** Reference patterns in mg/g protein from FAO reports on protein quality evaluation.

	FAO report 1991				FAO report 2013			Difference between “preschool children 1991” and “older than 3y 2013″
	Infant (0–1 y)	Preschool children (2–5 y)	Older children (10–12 y)	Adults	Infants (0–6 m)	Infants (6 m – 3 y)	Children (>3 y), adolescents, adults	
Histidine	26	**19**	19	16	21	20	**16**	3
Isoleucine	46	**28**	28	13	55	32	**30**	–2
Leucine	93	**66**	44	19	96	66	**61**	5
Lysine	66	**58**	44	16	69	57	**48**	10
Sulfur AA	42	**25**	22	17	33	27	**23**	2
Aromatic AA	72	**63**	22	19	94	52	**41**	22
Threonine	43	**34**	28	9	44	31	**25**	9
Tryptophan	17	**11**	9	5	17	8,5	**6,6**	5
Valine	55	**35**	25	13	55	43	**40**	−5

Bold values indicate the reference pattern recommanded for “adults”.

## Calculation of the chemical score

For each indispensable AA (IAA), the ratio between the AA content in the dietary protein and that in the reference pattern is calculated. A ratio above 1 signifies that the AA is present in sufficient quantities to satisfy the AA requirement. Among the ratios obtained for each of the nine IAAs, the lowest is retained as the chemical score which quantifies the degree of effects of the most limiting AA. Higher than 1, there is no limiting AA. Below 1, there is at least one limiting AA whose degree of insufficiency is reflected by this score. A score of 0.8 therefore means that the most limiting AA is 20% below the amount of this AA required in the target group of individuals. It may be noted that an increase of protein intake by 20% above the requirement could compensate this deficiency. Moreover, the scoring metric is a simplistic approach as it only reflects the ability of one dietary protein to satisfy *per se* the requirement, but in practice several protein sources compose the diet.

The choice of reference pattern is therefore a crucial factor in score calculation. The publication by Sa et al. (6) clearly showed the impact of the reference pattern used on the chemical score distribution for 1,200 lentil samples. For instance, the distribution of the ratios for sulfur AAs ranged from 0.6 to 0.83 for preschool children (i.e., profile 1991), 0.55 to 0.78 for 0.5–3 years (children) and 0.64 to 0.9 for 3 y and older (~adults). For tryptophan, these ranges were 0.63 to 0.75 for preschool children (FAO 1991), 0.84 to 0.97 for children and 1.08 to 1.25 for “adults”.

## Impact of the N to protein conversion factor

The conversion factor applied to extrapolate protein from nitrogen (N) has a marked impact on the chemical score. Indeed, the AA composition is determined in an ingredient or food and needs to be related to the mass of protein. To achieve this, one classic and universal strategy is to measure N and apply by default a conversion factor of 6.25. However, this factor overestimates the protein content of almost all protein sources. Specific factors exist for different protein sources and are more relevant (8), but from a regulatory point of view, a factor of 6.25 should be used. By overestimating the real protein content, this default conversion factor penalizes the chemical score. One compromise is to provide both values using both the default and specific factors. Another possible strategy is to sum up the amounts of AAs determined analytically, after correcting the mass by the hydration factor of free AAs vs. in-chain AAs, and to use this value as the true protein content of the ingredient or food. The first strategy of using both the by default and a specific conversion factor better ensures homogeneity among studies than the second strategy because inter-laboratory variability exists when measuring AAs. In particular, acid hydrolysis destroys a given proportion of AAs that might be heterogeneous in AAs, ingredients, laboratory conditions, etc. The accuracy of the correction applied to take account of this loss cannot be certified because no internal standards exist to control the hydrolysis yield.

The chemical score, i.e., the AA composition related to the reference pattern, is the principal determinant of a scoring quality index so that particular attention should be paid to this analytical component.

## Digestibility correction factors

To take account of the bioavailability of nitrogen or AAs, the chemical score can then be modulated by a digestibility factor. When corrected for whole protein (i.e., nitrogen) digestibility, the appropriate index is the PD-CAAS, which was recommended by the FAO in 1991. In their report, the experts stated that “for practical reasons, the rat balance method is the most suitable practical method for predicting digestibility by humans.” This is often interpreted as “digestibility must be measured at the fecal level in rats,” but in fact, if more accurate values have been obtained in pigs or humans at the ileal level, they can be used. Another interpretation of the PD-CAAS that could be discussed concerns the appropriate reference pattern. During the expert consultation in 1989, the reference pattern was established on the basis of the AA requirement in 1985 (preschool children, as referred to above). However, because AA requirements were markedly revised in 2007, and subsequently the reference pattern, it might be more logical to use the 2013 reference patterns to evaluate the PD-CAAS. In the same way as the N to protein conversion factor, the main reason put forward for using the 1991 reference pattern is regulatory.

In the 2011 expert consultation, the digestibility of each individual AA was proposed as the digestibility correction factor in place of protein digestibility. The main methodological difference between the DIAAS and PD-CAAS is that ileal values of AA digestibility are necessary, which is much more complex than measuring fecal protein digestibility. This challenge resulted in greater interest in the digestibility methodology and several alternative approaches, where *in vitro* (9) or minimally invasive *in vivo* (10), have been developed during the past decade. Another aspect that has been extensively debated is truncation of the PD-CAAS to 1 as this index was designed to reveal limiting AA but not to inform on excess AAs. It is however possible to indicate the non-truncated PD-CAAS, especially for comparisons with the DIAAS. The latter is not truncated, so that the ability of protein sources to offset each other can be acknowledged. Table 2 presents an internal comparison of these different scores for some protein sources.

**Table 2:** Chemical score, PD-CASS and DIAAS of protein sources assessed during clinical or pig studies.

	CS	Non-truncated PD-CAAS	DIAAS
Casein (10)	1.48 (SSA)	1.42	1.45 (SSA)
Whey (14)	1.08 (His)	0.99	1.03 (His)
Pea isolate (15)	1.06 (SSA)	0.98	1.00 (SSA)
Sunflower isolate (16)	0.99 (Lys)	0.85	0.86 (Lys)
Flaxseed isolate (11)	0.74 (Lys)	0.68	0.58 (Lys)
Faba beans (17)	0.78 (His, Trp)	0.66	0.66 (His, Trp)
Oat concentrate (18)	0.80 (Lys)	0.69	0.67 (Lys)
Soy flour (19)	0.97 (SSA)	0.93	0.89 (SSA)
Wheat (19)	0.56 (Lys)	0.51	0.45 (Lys)

For score calculation, protein content was determined using N x 6.25. Reference pattern used: individual >3 y (FAO 2013).

Table 2 reveals the relatively low impact of digestibility correction factors on the scores compared to the AA composition that is the main determinant of the quality scores. Moreover, one can notice the good consistency between DIAAS and PD-CAAS values, except for the study on flaxseed where a particularly low digestibility of the limiting AA (namely lysine) was observed; it was suspected to be ascribable to Maillard reactions in the food matrix, in that case a biscuit (11). As a result, a small difference between PD-CAAS and DIAAS values could be presumed for low processed ingredients or foods but greater discrepancies are probable for ultra-processed foods because specific AAs such as lysine or SSA are more sensitive to technological treatments. As for the issue of the reference pattern, if the FAO 1991 pattern for preschool children had been used to calculate the PD-CAAS, the latter would have been drastically lower; for instance 0.51 for Faba bean (Trp) or 0.7 for sunflower (Lys). This illustrates that the use of different reference patterns to compare PD-CAAS and DIAAS is biased, and the 2013 reference patterns for any quality score metrics should clearly be recommended in order to ensure consistency between the different quality indicators.

## Thresholds for claims regarding protein quality

Another novelty concerning the DIAAS metric was the proposal of thresholds in order to claim a good (DIAAS>0.75) or excellent (DIAAS>1) protein quality. Herreman et al. (12) reported DIAAS data on 17 protein sources, each involving several observations. Surprisingly, only casein and pork satisfied the criteria for an excellent source in both the older than 3 y and 0.5 y-3y patterns, but not in infants. A third of the sources, all from plants except gelatin (for which the DIAAS is null), did not reach the threshold for good quality, even under the >3y pattern. To appreciate the biological significance of this 0.75 threshold, it is necessary to compare DIAAS values with physiological markers of protein quality. In a recent review, Nosworthy et al. (13) collected values for DIAAS (using the 0.5–3 y reference pattern) and the Protein Efficiency Ratio (PER), which indicates the ability of protein to sustain growth in growing rats. The correlation between the two indexes was good (R = 0.84, *p* < 0.001) and all the products (except tofu) with a DIAAS value <0.75 had a low PER (<1.6), whereas a DIAAS higher than 1 was associated with a high PER. A more exhaustive collection of data may be necessary, especially for products with DIAAS values ranging from 0.75 to 1, but it appears from this rough analysis that a DIAAS score lower than 0.75 is associated with impaired growth.

## Conclusion

Quality scores are mainly dependent on the AA composition of the protein to which specific attention must be paid. The reference pattern applied, and determining the protein content of an ingredient or food, will also have a significant impact on quality scores. Digestibility correction factors have been complexified from PD-CAAS to DIAAS, resulting in a considerable growth of interest in digestibility methods. The technical challenges have been faced and interestingly, numerous data have been produced since the FAO report in 2013. DIAAS and PD-CAAS values are often very close because although some differences exist between N and individual AA digestibility, these correction factors exert limited influence on the quality scores, because the digestibility values of N and AA in various protein sources mostly range from 75 to 95%.
